# Global deletion of COX-2 attenuates hepatic inflammation but impairs metabolic homeostasis in diet-induced obesity

**DOI:** 10.1016/j.jlr.2025.100823

**Published:** 2025-05-08

**Authors:** Jeyakumar Balakrishnan, Cyrus Desouza, Rishikesh Thakare, Yazen Alnouti, Viswanathan Saraswathi

**Affiliations:** 1 Department of Internal Medicine, Division of Diabetes, Endocrinology, and Metabolism, University of Nebraska Medical Center, Omaha, NE, USA; 2 VA Nebraska-Western Iowa Health Care System, Omaha, NE, USA; 3 Department of Pharmaceutical Sciences, University of Nebraska Medical Center, Omaha, NE, USA

**Keywords:** COX-2, MASLD, obesity, inflammation, triglycerides, cholesterol

## Abstract

The role of cyclooxygenase-2 (COX-2), a well-known pharmacological target for attenuating inflammation, in regulating obesity and its comorbidities remains unclear. We sought to determine the role of COX-2 in modulating metabolic inflammation and systemic metabolic homeostasis in obesity. Male WT and COX-2 KO mice were fed a chow diet or a high fat diet (HF, 45% fat) for 13 weeks. While the body weight gain did not alter, the visceral adipose tissue mass was significantly higher in KO-HF mice than in WT-HF mice. Plasma triglycerides and total cholesterol levels were higher in KO-HF mice than in WT-HF mice. Total body fat mass was higher with a concomitant reduction in lean mass in KO-HF mice than in WT-HF mice. Paradoxically, hepatic steatosis was reduced in KO-HF mice. While liver triglycerides were reduced, the liver cholesterol was increased in KO-HF mice. Bile acids and markers of cholesterol biosynthesis were unaltered between WT-HF and KO-HF groups. The mRNA and/or protein levels of autophagy markers were significantly decreased in KO-HF mice compared to WT-HF mice, indicating that a reduction in autophagy may increase cholesterol levels in these mice. The liver inflammatory markers were significantly increased only in WT mice fed a HF diet but not in KO-HF fed mice compared to their respective controls. Visceral adipose tissue showed a reduction in inflammatory markers in spite of an increase in adiposity. These data suggest that despite being effective in attenuating the inflammatory processes, inhibition of COX-2 exerts undesirable consequences on metabolic homeostasis.

The incidence of metabolic dysfunction-associated steatotic liver disease (MASLD) and metabolic dysfunction-associated steatohepatitis (MASH) are increasing worldwide (1). This is due to an increase in the incidence of obesity and type 2 diabetes mellitus. Until recently, there were no approved drugs for MASH (2). However, resmetirom was approved by the FDA in 2024 as the first therapy specifically indicated for the treatment of MASH. However, patients taking this drug exhibit undesirable side effects including diarrhea and nausea (3). The etiology of MASH is highly complex, and mechanisms involved in the progression of MASH are not fully understood (4).

Cyclooxygenase (COX) is a key enzyme, involved in mediating an inflammatory response. All the nonsteroidal anti-inflammatory drugs act via inhibiting COX activity (5, 6). COX is required for the conversion of arachidonic acid to prostaglandins (PGs). Two COX isoforms have been identified, COX-1, the constitutive form, and COX-2, the inducible form. While the former is believed to be responsible for homeostasis, the latter is thought to exert actions mainly in pathological states (7). As MASLD progresses from simple steatosis to MASH and liver fibrosis, COX-2-derived PGs may contribute to inflammation. In fact, COX-2 expression and activity have been associated with obesity and MASLD (8, 9). Moreover, people with obesity or MASLD have high COX-2 levels in their adipose tissue (AT) and livers. These findings indicate a potential link between COX-2 and obesity and MASLD pathogenesis (10, 11) and suggest that inhibition of COX-2 may be an effective strategy in managing MASLD.

Of note, in addition to regulating inflammation, COX-derived PGs appear to play a role in altering lipid metabolism. Several lines of evidence suggest that COX-2 inhibitors attenuate hepatic steatosis in mice. For example, we previously reported that indomethacin, an isoform nonspecific COX inhibitor which inhibits both COX-1 and COX-2, attenuates hepatic steatosis and dyslipidemia in mice (12). Celecoxib, a COX-2 inhibitor, attenuated hepatic steatosis in AKT-triggered MASLD model (13). In addition, valdecoxib and nimesulide, other COX-2 inhibitors, attenuated hepatic lipid accumulation in high fat (HF) diet-fed mice (14, 15). However, the role of COX-2 in altering hepatic lipid metabolism is debatable. For example, exogenously added or endogenously stimulated PGs significantly improve lipid metabolism in arterial walls (16). Moreover, NS-398, a COX-2 inhibitor, downregulated cholesterol efflux proteins and promoted foam cell formation in macrophages (17). Celecoxib, another COX-2 inhibitor, was ineffective in attenuating hepatic steatosis in carbon tetrachloride and fructose-fed liver injury models (13, 18). Thus, the role of COX-2 in regulating hepatic lipid metabolism is still unclear. It should be noted that many of these studies were conducted using pharmacological agents to inhibit COX-2 activity which may exert off-target effects. The objective of this study is to define the role of COX-2 in altering hepatic steatosis and inflammation, features of obesity-related MASH, in genetically engineered mice lacking COX-2.

## Materials and Methods

### Mice and diet

COX-2^−/−^ mice on C57BL/6 genetic background were kindly provided by Dr Raymond Harris, Vanderbilt University, and were originally purchased from Jackson Laboratories. The WT C57BL/6 mice were obtained from Jackson Laboratories. The COX-2^−/−^ mice were extensively used to study the role of COX-2 in many diseases including hypertension, pancreatitis, and pulmonary fibrosis (19, 20, 21). We previously used these mice to study the role of COX-2 in myeloid cells in altering obesity-related metabolic disorders (22). Eight- to ten-week-old WT and COX-2^−/−^ (KO) mice were subjected to a chow diet (CD) or an HF diet containing 45% of the energy derived from fat (Research Diets Inc) for 13 weeks. All procedures involving the care of these animals were conducted in accordance with the approved guidelines from the Institutional Animal Care and Use Committee at the VA Nebraska-Western Iowa Health Care System in Omaha, Nebraska.

### Cell culture and reagents

Huh7 cells were grown in Dulbecco's modified Eagle's medium with high glucose (HyClone) and supplemented with 10% fetal bovine serum (Gibco) and 1% penicillin-streptomycin at 37°C with 5% CO2. NS398 was purchased from Caymen Chemicals and dissolved in DMSO. Chloroquine (CQ) was purchased from Sigma-Aldrich and was dissolved in phosphate-buffered saline.

### Metabolic studies

We recorded the weekly body weight and evaluated the body composition, which included lean and fat mass using an Echo MRI body composition analyzer. For studies on energy expenditure, mice were acclimatized for a period of 2 days and kept individually in Promethion cages supplied by Sable Systems International. We recorded the oxygen consumption (VO_2_), carbon dioxide production (VCO_2_), and food consumption for a span of 36 h. For insulin tolerance test (ITT) and glucose tolerance test (GTT), we fasted the mice for 5 h, and insulin (0.75 U/kg body weight) and glucose (1 g/kg body weight), respectively, were administered via intraperitoneal injection. We monitored the shifts in blood glucose levels at various intervals.

### Plasma measurements

We measured plasma total cholesterol and triglycerides (TGs) using commercial kits from Raichem. Blood glucose was measured using the Accu-chek Aviva glucometer.

### Bile acid analysis

Bile was collected from each gall bladder at sacrifice. The total sulfated and unsulfated bile acids were determined in bile as well as plasma samples using a highly sensitive, high-throughput liquid chromatography/mass spectrometry as we described previously (23, 24, 25, 26).

### Lipid analysis

The concentrations of TGs and cholesterol ester (CE) in the liver samples were quantified using gas chromatography (GC) at the Vanderbilt University Lipid Core Laboratory. Lipids were extracted using the method of Folch-Lees (27). The extracts were filtered and lipids recovered in the chloroform phase. Individual lipid classes were separated by thin layer chromatography using Silica Gel 60 A plates developed in petroleum ether, ethyl ether, acetic acid (80:20:1), and visualized by rhodamine 6G. TGs and CEs were scraped from the plates and methylated using BF3/methanol as described by Morrison and Smith (28). The methylated fatty acids were extracted and analyzed by GC. Gas chromatographic analyses were carried out on an Agilent 7890A gas chromatograph equipped with flame ionization detectors and a capillary column (SP2380, 0.25 mm × 30 m, 0.20 μm film, Supelco). Helium was used as the carrier gas. The oven temperature was programmed from 160°C to 230°C at 4°C/min. Fatty acid methyl esters were identified by comparing the retention times to those of known standards. Inclusion of lipid standards with odd chain fatty acids allowed quantitation of the amount of lipid in the sample. Trieicosenoin (C20:1) and cholesteryl eicosenoate (C20:1) were used as standards.

### RNA isolation and real-time PCR

We extracted total RNA from the liver and the visceral adipose tissue (VAT) using the TRIzol reagent supplied by Ambion, Life Technologies. The RNA was then reverse-transcribed to cDNA using the 5× iScript Reverse Transcription Supermix from Bio-Rad. We performed the real-time PCR to identify the mRNA levels of genes associated with lipid metabolism and inflammation. The real-time PCR was carried out using 1X iQ supermix (Biorad Laboratories), 2 μl of cDNA, and 1X Taqman primer-probes from Applied Biosystems (Table 1). To quantify gene expression, we used the ΔΔCT method and normalized the values to 18S ribosomal RNA.

**Table 1:** Primers for real-time PCR.

Gene (Abbr)	Description	Product #
*Acaca*	Acetyl-CoA carboxylase alpha	Mm01304273_m1
*Adgre1* (EMR-1; F4/80)	EGF-like module containing, mucin-like, hormone receptor like 1	Mm00802529_m1
*ApoE*	Apolipoprotein E	Mm01307193_g1
*ApoC III*	Apolipoprotein C-III	Mm00445670_m1
*Ccl2* (MCP-1)	Chemokine ligand 2/monocyte chemotactic protein 1	Mm00441242_m1
*Ccl3* (MIP-1a)	Chemokine ligand 3/macrophage inflammatory protein 1 alpha	Mm00441258_m1
*Cpt1*	Carnitine palmitoyltransferase 1	Mm00550438_m1
*Fasn*	Fatty acid synthase	Mm01253292_m1
*Hmgcr*	3-hydroxy-3-methylglutaryl-CoA reductase	Mm01282499_m1
*Cyp4a10*	Cytochrome P450, family 4, subfamily A, polypeptide 10	Mm01188913_g1
*Cyp7a1*	Cytochrome P450, family 7, subfamily A, polypeptide 1	Mm00484152_m1
*Cyp8b1*	Cytochrome P450, family 8, subfamily B, polypeptide 1	Mm00501637_s1
*Lep* (Leptin)	Leptin	Mm00434759_m1
*Tnfa*	Tumor necrosis factor, alpha	Mm00443258_m1

### Western blot analysis

Tissue samples and cell pellets were lysed in a buffer containing 20 mM Tris HCl, 150 mM NaCl, 1 mM EDTA, 1 mM EGTA, 0.5% NP-40, 2.5 mM sodium pyrophosphate, 1 mM sodium orthovanadate, and 1 mM glycerophosphate and proteinase inhibitor cocktail (Roche Diagnostics). Proteins were separated by SDS-PAGE and immunoblotted onto polyvinylidene fluoride membranes, and proteins of interest were detected using antibodies. The following proteins were detected using specific antibodies from Cell Signaling Technology: LC3A/B (Cat no:12741), ATG7 (Cat no:8558), P62 (Cat no:5114), PPARγ (Cat no:2443), F4/80 (Cat no:70076), TNFα (Cat no:11948), and cEPBα (Cat no:2295). Antibody against IL-1β (Cat no:26048-1-AP) was purchased from Protein Tech. GAPDH antibody (Cat no: MAB374) was from EMD Millipore. Protein bands were visualized using the Chemidoc Imaging System (Bio-Rad Laboratories).

### Histology

Liver and VAT samples were fixed in 10% formalin and paraffin blocks were prepared. The liver and VAT were cut into 4 μm and 7 μm sections, respectively, which were stained with H&E to determine the tissue morphology.

Liver and VAT sections were stained for F4/80, a macrophage marker, by immunohistochemistry. Briefly, deparaffinized tissue sections were incubated overnight with anti-F4/80 antibody (Cell Signaling Tech, MA) at 4°C. Sections were then incubated for 1 h with biotinylated secondary antibody (Vector IHC kit, Newark, CA) at room temperature. Vectastain ABC reagent (Avidin-Biotinylated HRP) was added for 30 min followed by incubation with peroxidase substrate 3,3′-diaminobenzidine, until color developed. Sections were counterstained with hematoxylin. Images (20X) were captured using the Leica light microscope.

### Confocal microscopy

For confocal microscopy, Huh-7 cells were plated in 6-well plates on a coverslip and treated with NS398 (20 μM) for 24 h followed by CQ at 10 μM concentration for 1 h. Cells were fixed with 4% paraformaldehyde, permeabilized using 0.2% Triton X-100, and incubated overnight with the LC3A/B antibody (Cell Signaling). Goat anti-rabbit Alexa 488-conjugated secondary antibody was used to visualize LC3II, and DAPI was used to stain the nuclei. Images were captured at 20X magnification using the Leica confocal microscope (Leica Microsystems).

### Statistical analysis

Data are expressed as mean ± SEM. Analysis was performed using the Prism GraphPad software 10.1. Statistical significance was determined by a 2-way ANOVA followed by Bonferroni *post-hoc* analysis for most of the experiments. Single factor comparisons were done by Student’s *t* test. For repeated measurements (e.g. ITT and GTT), a 2-way ANOVA was performed followed by Bonferroni’s *post-hoc* test and, by area under the curve. Energy expenditure was analyzed using the ANCOVA method. A *P*-value less than 0.05 was deemed to indicate statistical significance.

## Results

### Effect of COX-2 deletion in mice challenged with an HF diet on body weight and metabolic profile

**Fig. 1:**
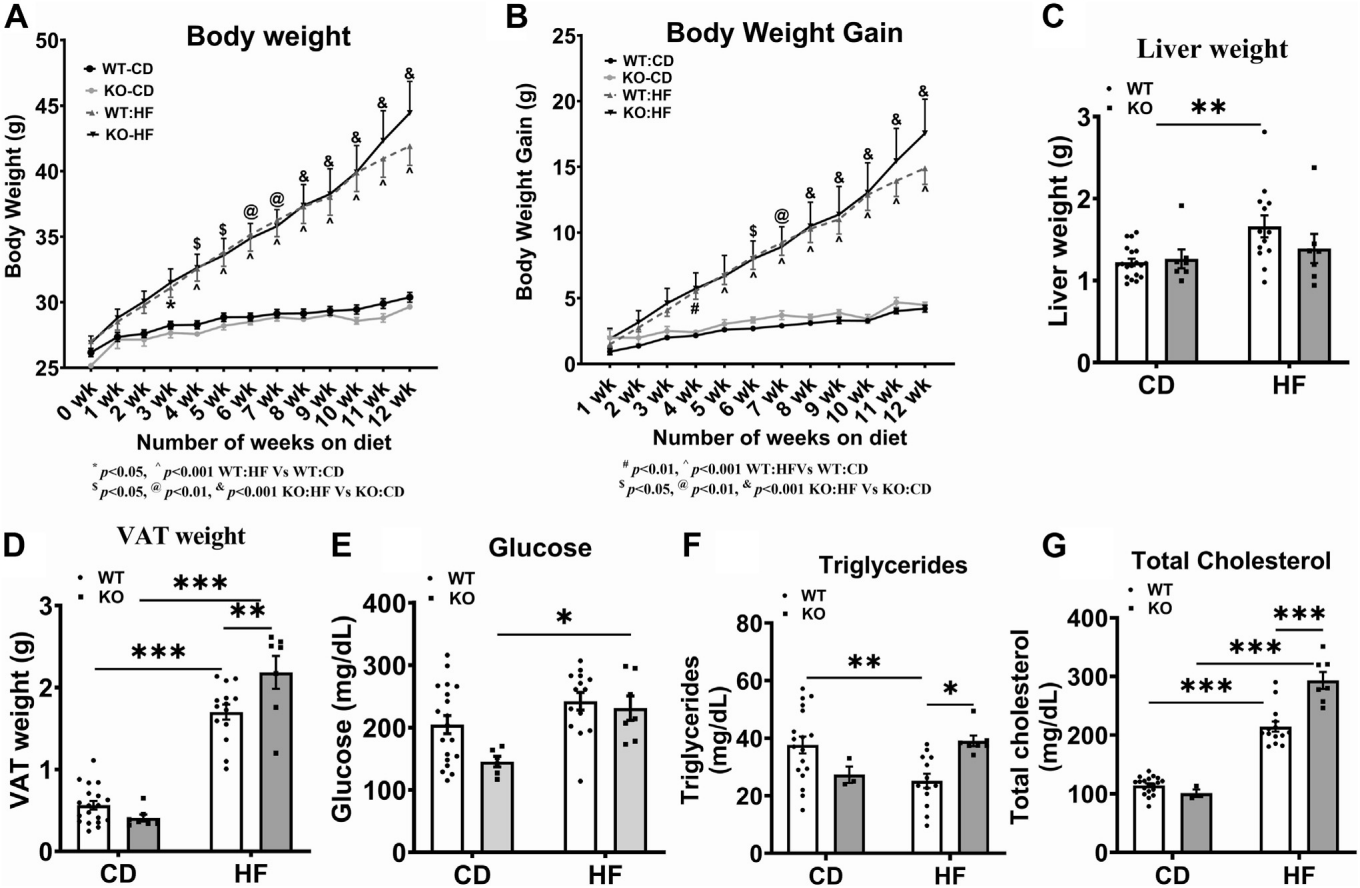
Effect of COX-2 deletion on metabolic variables in mice fed a high fat diet. Weekly body weight and body weight gain of mice were recorded (A and B). The liver and VAT weight were measured after euthanasia (C and D). Blood glucose (E), plasma triglycerides (F), and plasma total cholesterol (G) are shown. Values are expressed as mean ± SEM of 6–17 mice in each group. ^∗^*P* < 0.05, ^∗∗^*P* < 0.01, ^∗∗∗^*P* < 0.001. CD-Chow diet; HF-High fat; KO-Knockout; VAT, visceral adipose tissue; WT-Wild type.

We determined the body weight and body weight change from baseline in WT-CD, KO-CD, WT-HF, and KO-HF mice during the feeding period. Our data show that both WT and KO mice fed an HF diet exhibited an increase in total body weight as well as body weight gain compared to WT-CD and KO-CD mice, respectively (Fig. 1A, B). The body weight was not altered significantly between WT-HF and KO-HF groups. Moreover, the daily food intake did not alter between these two groups (not shown). The liver weight showed a significant increase only in WT-HF group but not in KO-HF group, compared to their respective controls (Fig. 1C). Interestingly, the VAT weight was significantly increased in both WT-HF and KO-HF mice, compared to their controls. Moreover, the VAT weight was significantly higher in KO-HF mice than in WT-HF group (Fig. 1D). Fasting blood glucose was significantly increased only in KO-HF compared to KO-CD group (Fig. 1E). Plasma TGs were significantly reduced in WT-HF mice, compared to WT-CD mice whereas its level did not alter between KO-CD and KO-HF groups. Of note, the KO-HF mice showed a significant increase in plasma TGs compared to WT-HF mice (Fig. 1F). Plasma TC was significantly increased in both WT-HF and KO-HF mice compared to their controls. Remarkably, the plasma TC levels were significantly elevated in KO-HF mice compared to WT-HF mice (Fig. 1G).

### Effect of COX-2 deletion on body composition and energy expenditure

**Fig. 2:**
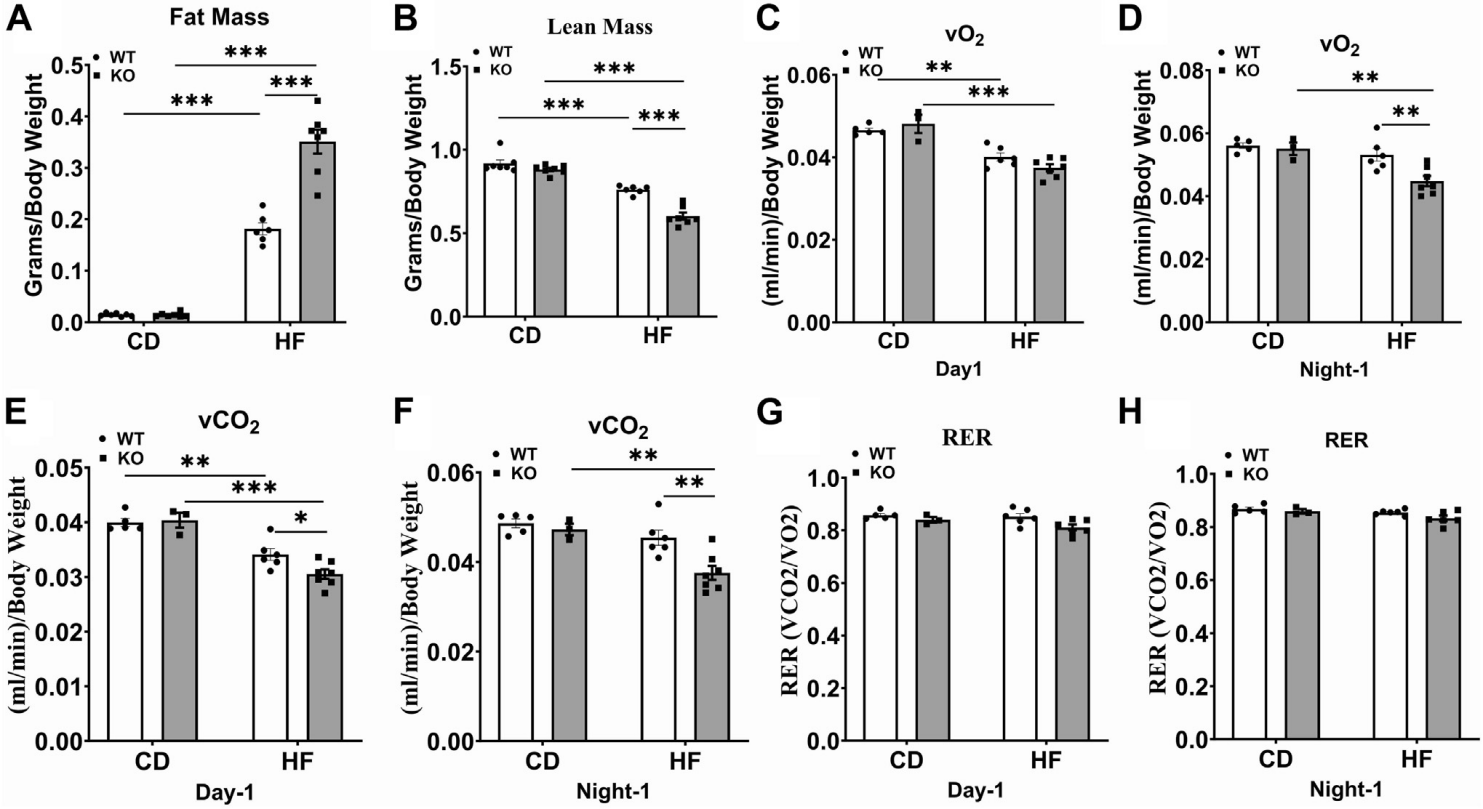
Effect of COX-2 deletion on body composition and energy expenditure in mice fed a high fat diet. The total fat mass (A) and lean mass (B) in live mice at 12 weeks postdiet are shown. Energy expenditure measured in terms of volume of oxygen consumed (C, D), carbon dioxide released (E, F), and RER ratio was determined (G, H). Values are expressed as mean ± SEM of 6–12 samples in each group. ^∗^*P* < 0.05, ^∗∗^*P* < 0.01, ^∗∗∗^*P* < 0.001. CD, chow diet; HF, high fat; RER, respiratory exchange ratio; vO2, volume of oxygen consumed; vCO2, volume of carbon dioxide released.

We measured the total fat mass and lean mass in WT and KO mice using the Echo-MRI body composition analyzer. The total fat mass was significantly higher in both WT-HF and KO-HF mice than their respective controls. Interestingly, we noted a significant increase in total fat mass in KO-HF mice compared to WT-HF group (*P* < 0.001) (Fig. 2A). The lean mass decreased significantly in WT-HF and KO-HF groups compared to controls. Moreover, it was significantly lower in KO-HF mice than in WT-HF mice (*P* < 0.001) (Fig. 2B). We next analyzed the energy expenditure using the metabolic cages. Our data show that the volume of oxygen consumed (vO_2_, Fig. 2C, D) and carbon dioxide released (vCO_2_, Fig. 2E, F) as normalized to body weight did not alter between WT-CD and KO-CD groups and were significantly lower in WT-HF and KO-HF mice than their respective controls on day 1 and/or night 1. In addition, we noted that vO_2_ and vCO_2_ were significantly lower in KO-HF mice than in WT-HF mice (Fig. 2D–F). However, adjusting the vO_2_ and vCO_2_ values to lean body mass by ANCOVA analysis showed that the KO-HF mice actually exhibited a higher energy expenditure than WT-HF mice on day 1. For example, on day 1, after adjusting for lean mass, KO mice had a higher model estimated mean vO_2_ than WT mice (1.31 and 1.54 in WT-HF and KO-HF groups, respectively, *P* = 0.004). No significant change was noted on night 1. The respiratory exchange ratio did not alter significantly among groups. The respiratory exchange ratio was around 0.8 in all groups, indicating that a mixture of carbohydrates and fat is used as energy fuel (Fig. 2G, H).

### Effect of COX-2 deletion on systemic glucose homeostasis

**Fig. 3:**
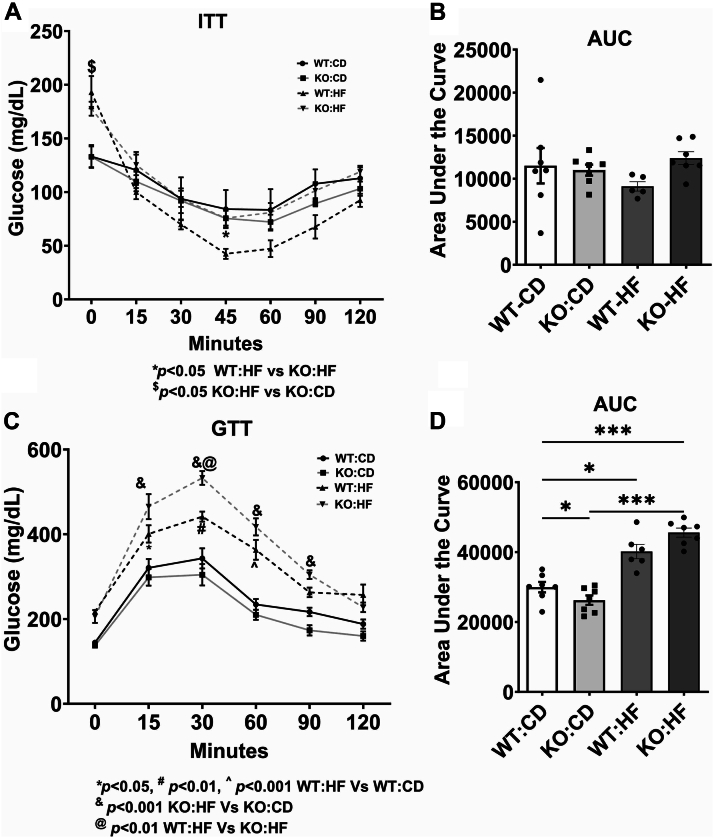
Effect of COX-2 deletion on systemic insulin resistance and glucose homeostasis in mice fed a high fat diet. Intraperitoneal insulin tolerance test was performed to assess systemic insulin resistance. Ten week postdiet, mice were injected (IP) with insulin at 0.75 U/kg body weight. Blood glucose levels were measured at indicated time points (A). The area under the curve was measured and plotted (B). Intraperitoneal glucose tolerance test was conducted to determine systemic glucose homeostasis. Eleven week postdiet, mice were injected (IP) with glucose at 1 g/kg body weight. Blood glucose levels were measured at indicated time points (C). The area under the curve was measured and plotted (D). Values are expressed as mean ± SEM of 6–8 samples per group. ^∗^*P* < 0.05, ^∗∗∗^*P* < 0.001. AUC, area under the curve; CD, chow diet; HF, high fat; ITT, insulin tolerance test; GTT, glucose tolerance test.

We next performed the ITT and GTT and our data show that the insulin resistance did not alter significantly among different groups (Fig. 3A, B). GTT revealed that the KO-CD mice showed a small but significant improvement in glucose response as analyzed by the area under the curve measurement (Fig. 3C, D). Both WT and KO mice on an HF diet exhibited a significant increase in glucose intolerance, compared to their controls whereas the degree of glucose intolerance was more in KO-HF group. However, no significant difference was noted between WT-HF and KO-HF groups.

### Effects of COX-2 deletion on liver lipid metabolism

**Fig. 4:**
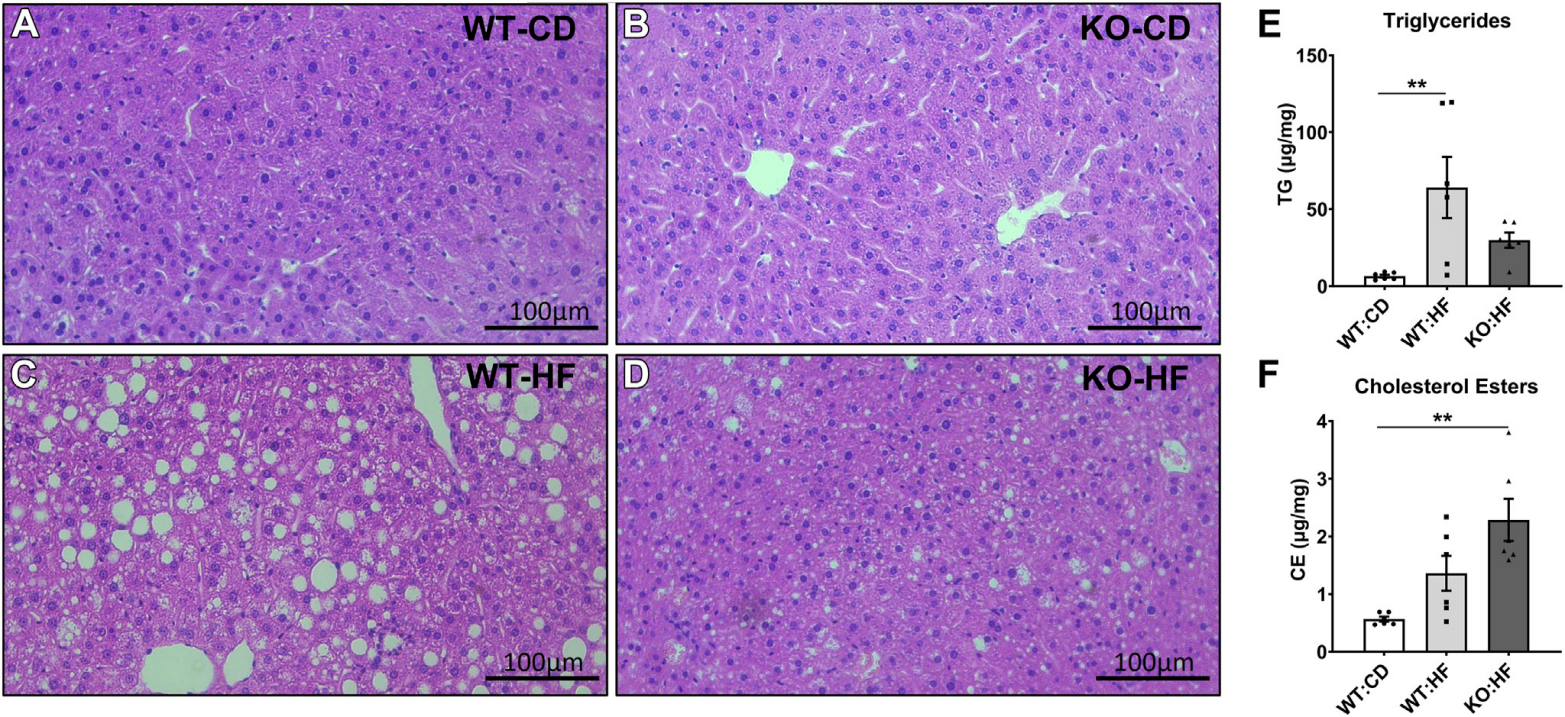
Effect of COX-2 deletion on liver lipid metabolism. Representative photographs of H&E-stained liver sections at 20X magnification captured using the Leica light microscope are shown for WT-CD (A), KO-CD (B), WT-HF (C), and KO-HF (D) groups. Liver triglycerides (E) and cholesterol esters (F) were measured by gas chromatography. Values are expressed as mean ± SEM of 6–8 samples in each group. ^∗∗^*P* < 0.01. CD, chow diet; HF, high fat.

To determine whether HF feeding leads to MASLD, we first performed an H&E staining of liver sections. Both WT-CD and KO-CD groups did not exhibit steatosis (Fig. 4A, B). We noted a profound increase in steatosis in WT-HF mice (Fig. 4C) compared to WT-CD (Fig. 4A). The KO-HF mice also showed an increase in steatosis but to a lesser extent compared to WT-HF group (Fig. 4D). We next measured the liver TGs by GC and found that the liver TG levels were significantly elevated only in WT-HF mice (*P* < 0.01) but not in KO-HF group, compared to WT-CD mice (Fig. 4E). Although the liver TGs did not increase significantly in KO-HF mice compared to WT-CD group, the liver CEs showed a significant increase (*P* < 0.01). However, despite a profound increase in liver TGs, the CE accumulation did not alter significantly in WT-HF mice, compared to WT-CD mice (Fig. 4F).

**Fig. 5:**
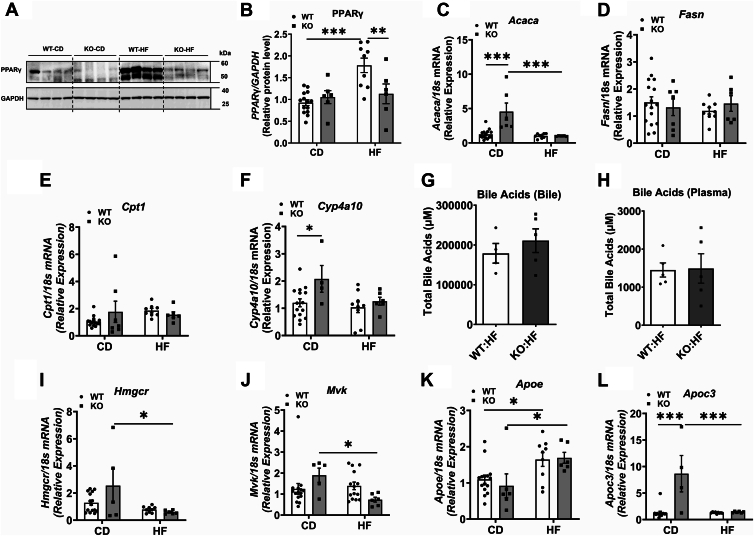
Effect of COX-2 deletion on markers regulating liver lipid metabolism. Western blot analysis of PPARγ protein levels in liver with representative images (A). After densitometric analysis of band intensities, values are normalized to GAPDH and expressed as mean ± SEM of 6–8 samples per group (B). Real-time PCR analysis was carried out in liver samples for the mRNA levels of genes altering fatty acid synthesis (C, D) and fatty acid oxidation (E, F). Bile acids (G, H), mRNA levels of genes altering cholesterol biosynthesis (I, J), and lipoprotein secretion (K, L) are also shown. For real-time PCR analysis, values are normalized to 18S and expressed as mean ± SEM of 6–12 samples per group. ^∗^*P* < 0.05, ^∗∗^*P* < 0.01, ^∗∗∗^*P* < 0.001. CD, chow diet; HF, high fat.

To determine whether COX-2 deletion alters hepatic lipid metabolism, we first measured the protein level of PPARγ, a key player in lipid droplet formation, in the liver. We noted that PPARγ is significantly higher in WT-HF mice than in WT-CD mice. On the other hand, a significant decrease in PPARγ was noted in KO-HF, compared to WT-HF group (Fig. 5A, B). We next measured the mRNA levels of genes regulating liver lipid metabolism. The mRNA levels of *Acaca*, in KO-CD mice significantly increased compared to WT-CD mice (Fig. 5C), whereas in HF-fed mice, the lipid overload suppresses *Acaca* mRNA levels regardless of the presence or absence of COX-2. The mRNA level of *Fasn* was not significantly altered among different groups (Fig. 5D). Regarding fatty acid oxidation, the mRNA level of *Cpt1* did not alter among groups (Fig. 5E). *Cyp4a10*, another gene involved in fatty acid oxidation, increased significantly in KO-CD mice compared to WT-CD mice. However, no difference was noted between WT-HF and KO-HF groups (Fig. 5F). Overall, while the role of altered fatty acid synthesis and/or fatty acid oxidation in reducing liver TG accumulation in the liver of KO-HF mice is unclear, these data suggest that the reduction in PPARγ could play a role at least in part, in mediating this effect.

As mentioned, we noted an increase in CE levels in KO-HF mice compared to WT-CD mice (Fig. 4F). The plasma TC level was also significantly higher in KO-HF mice than in WT-HF group (Fig. 1G). Cholesterol conversion to bile acids is an important mechanism by which excess cholesterol is eliminated from the body. Thus, the increase in liver and plasma cholesterol can be attributed to a reduction in cholesterol metabolism to bile acids. To test this notion, we analyzed the total bile acid levels in gall bladder bile and plasma and noted that it did not vary between WT-HF and KO-HF groups (Fig. 5G, H). Another potential mechanism for the increase in hepatic and plasma cholesterol is increased endogenous cholesterol production in the liver. To test this possibility, we analyzed the mRNA levels of genes involved in cholesterol biosynthesis. Our data show that the mRNA levels of *Hmgcr*, a gene encoding for the rate-limiting enzyme in cholesterol biosynthesis, *Mvk*, another gene involved in endogenous cholesterol synthesis, were rather reduced in KO-HF mice compared to KO-CD mice (Fig. 5I, J). These data indicate that the increase in hepatic and plasma cholesterol in KO-HF mice may not be due to an increase in hepatic cholesterol biosynthesis. Finally, we analyzed the mRNA levels of lipoproteins, and we noted that the mRNA level of *Apoe* increased significantly in both WT-HF and KO-HF groups, compared to their CD controls (Fig. 5K). Interestingly, the mRNA level of *Apoc3* was significantly increased in KO-CD mice compared to WT-CD mice. On the other hand, the HF diet inhibited *Apoc3* mRNA in both WT and KO mice (Fig. 5L). Regardless, neither *Apoe* nor *Apoc3* alter significantly between WT-HF and KO-HF groups (Fig. 5K, L). It is unclear whether altered lipoprotein secretion contributes to an increase in plasma cholesterol levels in KO-HF mice, but this possibility cannot be ruled out.

### Effects of COX-2 deletion on liver autophagy

**Fig. 6:**
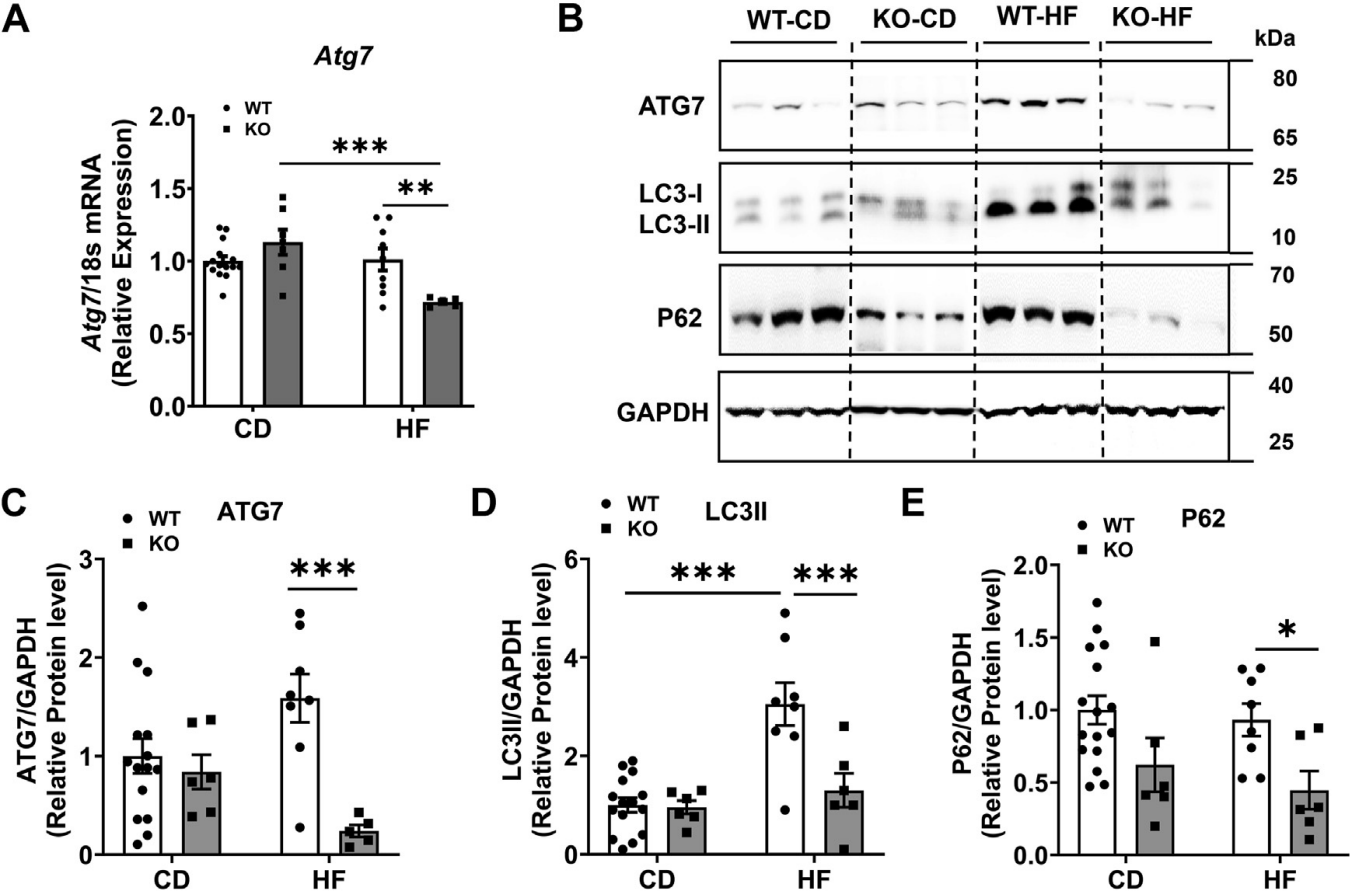
Effect of COX-2 deletion on markers of autophagy. Real-time PCR analysis was carried out in liver samples for the mRNA levels of ATG7 (A). Values are normalized to 18S and expressed as mean ± SEM of 6–12 samples per group. Western blot analysis of indicated autophagy-related protein levels in liver with representative images (B). After densitometric analysis of band intensities, values are normalized to GAPDH and expressed as mean ± SEM of 6–16 samples per group (C–E). ^∗^*P* < 0.05, ^∗∗^*P* < 0.01, ∗∗∗*P* < 0.001. CD, chow diet; HF, high fat.

Autophagy is another mechanism by which cholesterol homeostasis is regulated. We next analyzed the mRNA levels of autophagy markers and noted a significant reduction in *Atg7* in KO-HF mice compared to KO-CD and WT-HF mice (Fig. 6A). Further Western blot analysis for the protein levels of autophagy markers revealed that ATG-7 protein level was significantly lower in KO-HF when compared to WT-HF group (Fig. 6B, C). Moreover, the protein levels of LC3II which is responsible for the formation of autophagosomes were significantly higher in WT-HF mice than in WT-CD mice. However, the KO-HF mice did not show an increase in LC3II compared to KO-CD mice. On the other hand, the LC3II protein level was significantly lower in KO-HF mice when compared to WT-HF mice (Fig. 6B, D). Also, the western blot analysis for the protein level of P62, which acts as a selective autophagy cargo receptor, clearly revealed that the P62 protein level was significantly lower in KO-HF mice than in WT-HF mice (Fig. 6B, E). Taken together, these results suggest that the deletion of COX-2 leads to impaired/dysregulated autophagic flux in liver which can lead to increased cholesterol accumulation in liver and plasma.

**Fig. 7:**
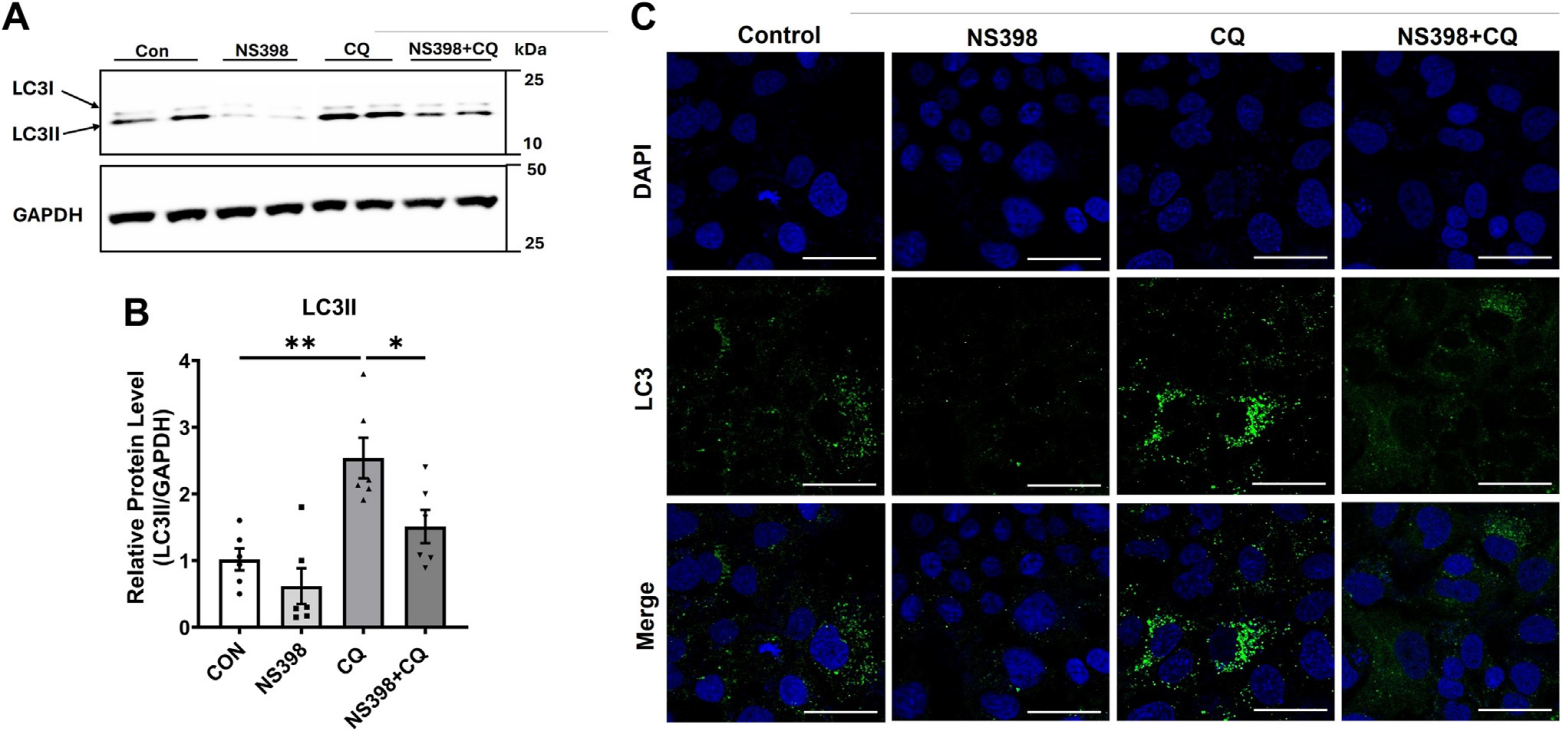
Pharmacological inhibition of COX-2 attenuates autophagy in Huh-7 cells in vitro. Huh-7 cells were treated with or without 20 μM of NS-398 for 24 h followed by 1 h of 10 μM chloroquine (CQ). LC3II was examined by Western blotting. GAPDH was used as an internal control. Representative Western blot images are shown (A). Densitometric analysis of band intensities is shown (B). Data are presented as mean ± SEM of three samples per group. ^∗^*P* < 0.05 and ^∗∗^*P* < 0.01. Huh-7 cells were treated with NS-398 (20 μM) for 24 h followed by CQ (10 μM) for 1 h and examined for LC3II by immunofluorescence assay using confocal microscopy (C). Images were captured at 20X magnification using Leica confocal microscope. A representative picture from each group is shown (n = 3 per group).

To further ascertain the role of COX2 in altering autophagy, we performed experiments in cultured cells. We treated Huh-7 hepatoma cells with CQ, an autophagy inhibitor, in the presence or absence of NS398, a selective COX2 inhibitor. Cells treated with NS398 alone showed a trend towards a decrease in LC3II accumulation. As expected, CQ significantly increased LC3II accumulation whereas cells treated with both NS398 and chloroquine showed a significant reduction in LC3II, compared to cells treated with CQ alone (Fig. 7A, B). Similarly, confocal microscopy results also showed that CQ treatment increased the LC3II puncta staining in Huh-7 cells. Cells treated with NS-398+CQ showed less punctate staining compared to CQ alone-treated cells (Fig. 7C), indicating a reduction in autophagy flux.

### Effects of COX-2 deletion on liver inflammatory markers

**Fig. 8:**
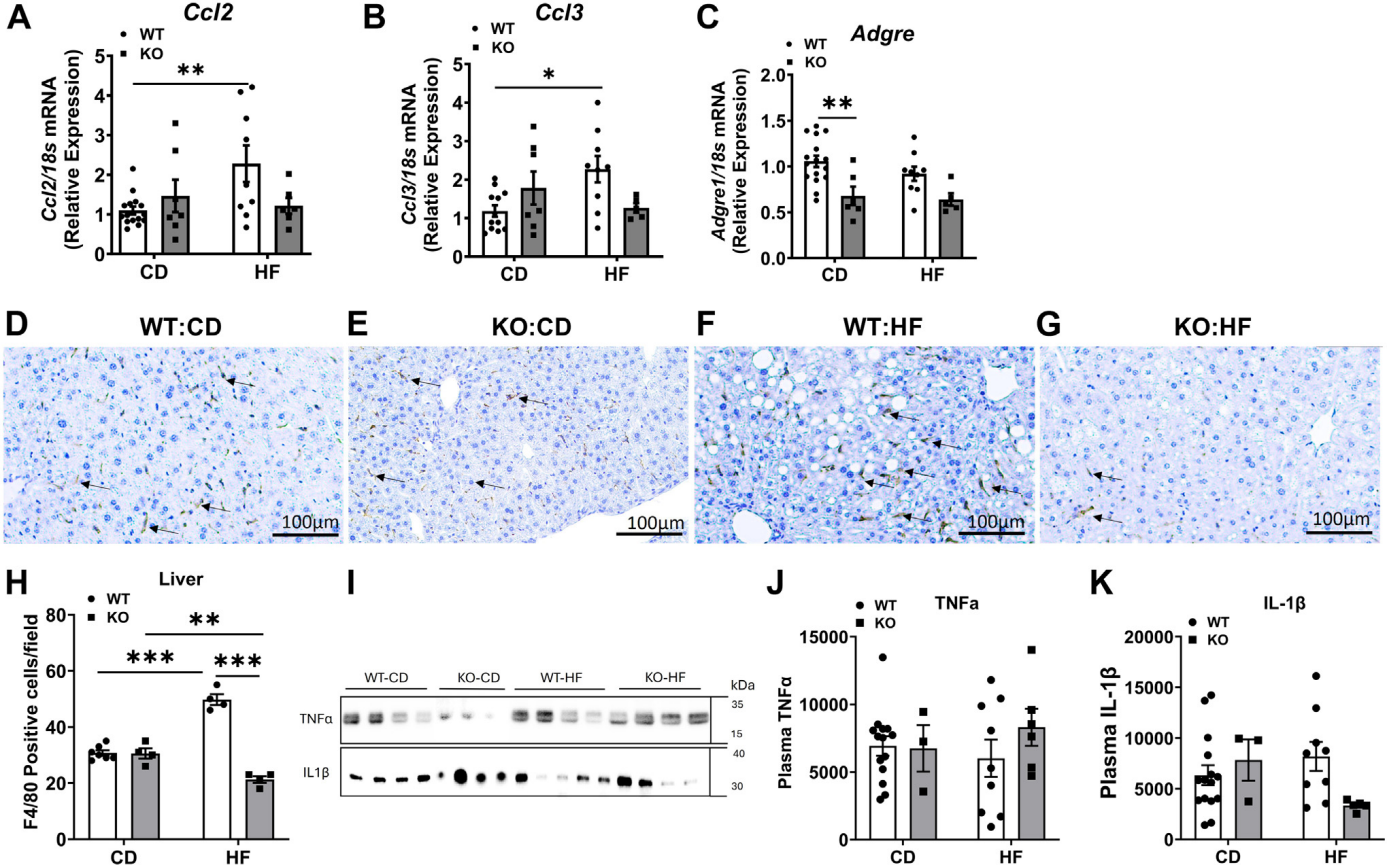
Effect of COX-2 deletion on liver inflammatory markers in mice fed a high fat diet. Real-time PCR analysis was carried out in liver samples for the mRNA levels of inflammatory genes and a macrophage marker (A–C). Values are normalized to 18s and expressed as mean ± SEM of 6–12 samples in each group. Immunostaining for F4/80, a macrophage marker, in liver sections from WT-CD (D), KO-CD (E), WT-HF (F), and KO-HF (G) was carried out. Images were captured at 20X magnification. A representative picture from each group is shown. Quantification of liver macrophage numbers per field (H). Values are expressed as mean ± SEM of 4–7 samples per group. Western blot analysis of inflammatory markers TNFα and IL-1β in plasma was carried out and representative images are shown (I). Band intensities were quantified by densitometric analysis and values are expressed as mean ± SEM of 6–8 samples per group (J and K). ∗*P* < 0.05, ^∗∗^*P* < 0.01, ^∗∗∗^*P* < 0.001. CD, chow diet; HF, high fat.

We next studied the effect of COX-2 deletion on hepatic inflammatory markers. The mRNA levels of *Ccl2* and *Ccl3* were not significantly altered between WT-HF and KO-HF mice. However, only the WT-HF but not the KO-HF mice showed a significant increase in these mRNA levels, compared to their respective controls, indicating that markers of inflammation were blocked in KO-HF mice (Fig. 8A, B). The mRNA level of *Adgre1*, encoding F4/80, *a* macrophage marker, was significantly lower in KO-CD mice (*P* < 0.01) when compared with WT-CD mice (Fig. 8C). However, it did not alter in KO-HF mice compared to WT-HF mice. We next performed the immunohistochemical analysis for F4/80 in liver sections to detect the presence of macrophages. We found that the F4/80 staining did not alter between WT-CD and KO-CD groups (Fig. 8D, E). The F4/80 staining was remarkably higher in WT-HF mice (Fig. 8F) than in WT-CD mice. On the other hand, the KO-HF mice showed a marked reduction in F4/80 staining (Fig. 8G). Quantification of F4/80^+^ cells showed a significant increase in WT-HF mice, compared to WT-CD group. The number of F4/80^+^ cells was significantly lower in KO-HF mice than in WT-HF mice (Fig. 8H). The plasma levels of inflammatory markers, TNFα and IL1β, did not alter significantly among groups (Fig. 8I, K). Altogether, our data suggests that COX-2 deletion improves HF diet-induced liver inflammation in mice.

### COX-2 gene deletion attenuates HF diet-induced adipose tissue inflammation in mice

**Fig. 9:**
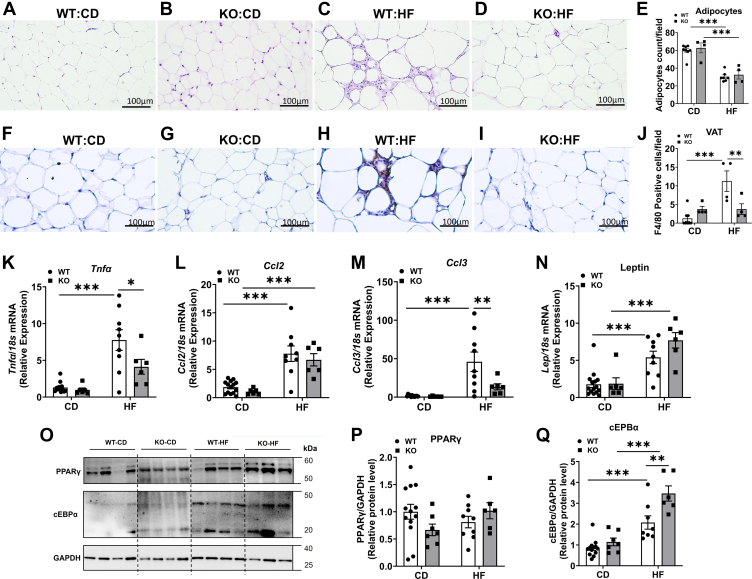
Effect of COX-2 deletion on inflammatory markers in visceral adipose tissue. VAT sections from WT-CD (A), KO-CD (B), WT-HF (C), and KO- HF (D) were stained with H&E. Representative images from each group at 20X magnification are shown. Quantification of adipocyte numbers per field (E). Values are expressed as mean ± SEM of four samples per group. Immunostaining for F4/80 in VAT sections from WT-CD (F), KO-CD (G), WT-HF (H), and KO-HF (I) was performed. Representative images from each group at 20X magnification are shown. Quantification of AT macrophage numbers per field (J). Values are expressed as mean ± SEM of 4–6 samples per group. Real-time PCR analysis was carried out in VAT for the mRNA levels of inflammatory genes and adipokines (K–N). Values are expressed as mean ± SEM of 6–12 samples per group. Western blot analysis for PPARγ and cEPBα was performed in VAT samples and representative images are shown (O). Densitometric analysis of band intensities normalized to GAPDH (P, Q). Values are expressed as mean ± SEM of 6–12 samples per group. ^∗^*P* < 0.05, ^∗∗^*P* < 0.01, ^∗∗∗^*P* < 0.001. CD, chow diet; HF, high fat.

To determine whether COX-2 deletion inhibits VAT inflammation, we first performed an H&E staining on VAT sections. We noted an increase in immune cell infiltration in the WT-HF mice. Interestingly, the immune cell infiltration was remarkably reduced in KO-HF mice (Fig. 9A–D). In addition, we noted a decrease in adipocyte numbers in WT-HF and KO-HF mice, compared to their controls (Fig. 9E), which could be attributed to an increase in adipocyte size due to increased fat accumulation in these groups. The immunohistochemical analysis of VAT sections for F4/80, a macrophage marker, showed a profound increase in F4/80 staining in WT-HF mice but not in KO-HF mice compared to their respective controls (Fig. 9F–I). Quantification of AT macrophage numbers showed a significant increase in WT-HF mice compared to WT-CD group. On the other hand, the macrophage number did not alter between KO-CD and KO-HF groups. Moreover, the number of macrophages was significantly lower in KO-HF mice than in WT-HF mice (Fig. 9J). We further measured the VAT inflammatory markers by RT-PCR analysis. Our data revealed that the mRNA levels of *Tnfα* (*P* < 0.001), *Ccl2* (*P* < 0.01), and *Ccl3* (*P* < 0.001) were significantly higher in WT-HF mice than in WT-CD mice (Fig. 9K–M). On the other hand, *Tnfα* (*P* < 0.05) and *Ccl3* (*P* < 0.01) mRNA levels were significantly lower in KO-HF mice compared with WT-HF mice (Fig. 9K, M). Further, we analyzed the mRNA levels of adipokines and noted that the *adiponectin* mRNA did not alter significantly among different groups (not shown). The *leptin* mRNA level showed a significant increase in WT-HF and KO-HF mice, compared to their controls (*P* < 0.001) (Fig. 9N). We analyzed markers of adipogenesis. Our data show that PPARγ protein level was not altered significantly among different groups (Fig. 9O, Q). Interestingly, cEBPα, another adipogenic marker, showed a significant increase in both WT-HF and KO-HF mice compared to their controls (Fig. 9O, Q). Of note, the KO-HF mice showed a significant increase in cEBPα, compared to WT-HF mice. Taken together, these results suggest that COX-2 deletion attenuates macrophage infiltration and inflammatory response in the adipose tissue upon an HF diet feeding. Our data also suggest that COX-2 deletion leads to increased adipogenesis and fat storage in AT.

## Discussion

In summary, we have demonstrated that mice lacking COX-2, fed an HF diet, exhibited an increase in fat mass and VAT mass, compared to WT-HF mice. Moreover, the KO-HF mice had an increase in plasma TC level. Intriguingly, hepatic steatosis and inflammation were lower in KO-HF mice. While the liver TGs were lower, the liver CE level was significantly higher with a concomitant reduction in autophagy markers, in particular, ATG7, LC3II, and P62 in KO-HF mice. Analysis of VAT revealed that markers of AT inflammation were lower despite an increase in adiposity. Taken together, our data suggest that deletion of COX-2 leads to a reduction in hepatic and adipose inflammation but an impairment in metabolic homeostasis.

An interesting finding of this study is that deleting COX-2, an inflammatory gene, leads to a profound impairment in metabolic homeostasis. Although COX-2 is considered to be an inducible enzyme whose level is increased by inflammatory stimuli, it is also present constitutively in many organs including the brain, uterus, and kidneys, indicating that it plays an important role in regulating the physiological processes as well (8, 29, 30). However, the role of COX-2 in regulating the metabolic processes in obesity remains unclear. Our study demonstrates that ablation of COX-2 results in increased adiposity and dysregulated cholesterol metabolism on an HF diet feeding, suggesting that COX-2 plays an integral role in regulating metabolic homeostasis. Adipocyte-specific COX-2 deletion has been previously shown to result in increased adiposity, accompanied by impaired glucose and insulin tolerance, with no significant changes in lean mass (31). In our global COX-2-KO model, we show that COX-2 deletion results in impairments in glucose homeostasis as analyzed by GTT. The GTT revealed that the KO-HF mice exhibited a more severe glucose intolerance as opposed to WT-HF mice, when compared to their respective controls. The lean mass was also significantly lower in KO-HF mice, compared to WT-HF mice, indicating that the loss of lean mass can partly contribute to the impaired glucose homeostasis. A previous study has shown an increase in circulating cholesterol levels and tissue cholesterol accumulation with COX-2 inhibition or deletion via disruptions in cholesterol efflux (32). Our study corroborates these findings and shows that cholesterol levels are increased in liver and plasma. Our data provide further evidence that although cholesterol levels are increased in liver, the TG accumulation is reduced in the absence of COX-2. Interestingly, our data show that the plasma TGs were significantly increased in KO-HF group compared to WT-HF mice. Of note, the plasma TGs were significantly lower in WT-HF mice than in WT-CD mice. Our finding is in line with a previous study showing that an HF diet feeding leads to a decrease in plasma TGs (33). This could be attributed to an increase in AT expansion as well as the storage of TGs in the liver in the HF-fed mice. Taken together, these data suggest that the KO-HF mice exhibit an impairment in systemic lipid homeostasis.

Regarding energy expenditure, a previous report showed that overexpression of COX-2 in white AT induced browning of white AT, increased systemic energy expenditure, and protected against obesity (34). Accordingly, in our study, the KO-HF mice exhibited a reduction in energy expenditure, compared to WT-HF mice, when normalized to body weight. However, adjusting energy expenditure to lean body mass showed that the KO-HF mice actually exhibited an increase in energy expenditure. Thus, the impact of COX-2 on energy expenditure is still unclear.

Another interesting finding is that the KO mice exhibit a reduction in hepatic steatosis with a concomitant decrease in hepatic inflammation. Evidence suggests that COX-2 inhibition is effective in attenuating MASLD in mice (35). For example, nimesulide, a cyclooxygenase-2 selective inhibitor, suppresses obesity-related MASLD through the regulation of peroxisome proliferator-activated receptor γ (15). As mentioned, other types of COX-2 inhibitors including celecoxib and valdecoxib are also effective in attenuating MASLD (13, 14, 36). Our data corroborates with these findings and show that the deletion of COX-2 attenuates hepatic steatosis. It should also be pointed out that COX-2 deletion while attenuating hepatic steatosis promotes adiposity, indicating that the hepatic TGs are directed to AT for storage in these mice. While TG is a primary lipid species increased in MASLD liver, CE levels are also increased in the liver of MASLD mice. Our data show that the hepatic CE content was in fact higher in KO-HF mice. Of note, this was associated with an increase in plasma TC levels in KO-HF mice. Together, these data suggest that deleting COX-2 is effective in attenuating hepatic TGs but leads to an impairment in cholesterol homeostasis.

Evidence suggests that COX-2 has a role in regulating cholesterol metabolism. For example, NS-398, a COX-2 inhibitor, downregulated cholesterol efflux proteins and promoted foam cell formation in macrophages (17). Celecoxib has been shown to interfere with cholesterol outflow from macrophages (37). In fact, disruption of cholesterol efflux by COX inhibitors is linked to their increased cardiovascular risk (38). While the role of COX-2 in altering cholesterol efflux from macrophages has been recognized, its role in altering hepatic cholesterol metabolism remains unknown. Our data show that hepatic CE and plasma TC were increased in KO-HF mice compared to WT-HF mice. An increase in cholesterol can be due to an increase in endogenous cholesterol synthesis or a reduction in cholesterol conversion to bile acids. However, the mRNA levels of *Hmgcr* and *Mvk*, enzymes involved in cholesterol synthesis, were not altered between WT-HF and KO-HF mice. Moreover, we did not see any change in the total bile acids in gall bladder bile and plasma between the WT-HF and KO-HF groups. Thus, the role of these processes in altering cholesterol levels in KO-HF mice is unclear. Of note, autophagy plays a role in promoting the clearance of lipid droplet-associated CEs in macrophages (39). Our data show that the mRNA level of *Atg7*, a gene involved in autophagy, was significantly lower in KO-HF group, compared to WT-HF group. ATG7 plays a crucial role in lipophagy and it promotes autophagosome formation (40). Western blot analysis revealed a reduction in ATG7 and LC3II levels in COX-2 KO mice, indicating a defective autophagy. In fact, a decrease in ATG7 leads to impairments in lipophagy and an increase in lipid accumulation (41). The low p62 levels seen in KO-HF mice could also indicate reduced autophagosome formation. Our in vitro studies in Huh-7 cells further provide evidence that inhibition of COX-2 inhibits autophagy. These data suggest that impaired autophagy could play a role, at least in part, in promoting hepatic cholesterol accumulation and/or increased plasma cholesterol levels in the absence of COX-2.

It should be pointed out that in addition to hepatocytes, other liver cells can also contribute to the reduction in hepatic TG levels in KO-HF mice. A cross-talk between hepatic macrophages and hepatocytes promotes hepatic steatosis. For example, macrophage M1 polarization and TLR4 activation and cytokines derived from macrophages can promote steatosis in hepatocytes [reviewed in (42)]. Our data clearly show that the number of F4/80^+^ macrophages are less in KO:HF mice, indicating that the reduction in macrophages and/or their inflammatory response in the absence of COX-2 can also partly contribute to the reduction in hepatic TG accumulation. Hepatic stellate cells can also play a role in promoting fat storage in hepatocytes. The chemokine secreted from activated hepatic stellate cells (HSC) directly induces steatosis in hepatocytes (43). COX-2 is highly expressed in HSCs and therefore, the role of altered HSC function in the absence of COX-2 in attenuating TG storage in liver remains possible.

Finally, our data show that the total fat mass and VAT mass are higher in KO mice, compared to WT mice on an HF diet. Interestingly, the protein level of cEBPα was significantly higher in the VAT of KO-HF mice, indicating that adipogenesis is increased in the absence of COX-2. These data suggest that partitioning of TGs from liver to adipose tissue can also contribute to TG reduction in the liver of KO mice. We previously demonstrated that deletion of COX-2 only in myeloid cells promotes adiposity (22). Moreover, our current findings are in line with another study showing that overexpression of COX-2 reduces adiposity (34). Together, these studies suggest that COX-2 deletion can increase adiposity. However, the VAT levels of macrophage and inflammatory markers are lower in KO-HF mice than in WT-HF mice. The reduction in VAT inflammation is consistent with the role of COX-2 in promoting an inflammatory response. However, it is not associated with an improvement in metabolic homeostasis.

Our findings are clinically relevant. For example, COX-2 inhibitors are known to exhibit cardiovascular side effects (44, 45). Disruption of cholesterol metabolism in macrophages and arterial walls are known to have a role in promoting adverse cardiovascular side effects. Our data suggest that blocking COX-2 activity can also impair hepatic cholesterol metabolism. Next, cancer cachexia characterized by a reduction in fat and muscle mass (46, 47) impairs the treatment response in cancer patients. In fact, celecoxib, a COX-2 inhibitor has been used clinically in cancer patients as an anti-inflammatory agent and showed benefit against cachexia and improved lean mass (48). Although the lean mass is reduced in KO mice in our study, our data suggest that administration of a pharmacological agent to inhibit COX-2 may still be helpful to improve cancer cachexia by increasing fat mass.

A few studies using a genetic approach showed differential effects of COX-2 in altering liver pathology. For example, Hu *et al.* showed that inhibition of COX-2 using COX-2 shRNA inhibited the proliferation of activated hepatic stellate cells and reduced fibrotic markers in rat liver (49). Along the lines, a study by Yu *et al.* showed that transgenic overexpression of human COX-2 in mouse hepatocytes resulted in liver-specific inflammatory disease (50). On the other hand, Motino *et al.* showed that overexpression of human COX-2 in mouse hepatocytes attenuated MASLD (51). It is unclear why COX-2 overexpression exerts differential effects on MASLD progression. One plausible reason could be due to the strategy used in these two studies in creating transgenic mice. Yu *et al.* overexpressed human COX-2 using the liver-specific transthyretin promoter whereas Motino *et al.* overexpressed human COX-2 in mouse hepatocytes under the control of the human ApoE promoter and its specific hepatic control region, a unique regulatory domain that directs ApoE expression in the liver. Regardless, using COX-2 global KO mice on a C57BL/6 background, the widely used mouse strain in obesity studies, we provide evidence that ablation of COX-2 attenuates hepatic inflammation and TG accumulation but impairs hepatic cholesterol metabolism. Additionally, we show that COX-2 KO mice exhibit a preferential fat accumulation in the adipose tissue.

Taken together, our data suggest that COX-2, an inflammatory gene, has a critical role in altering the metabolic processes. Although COX-2 deletion attenuates HF diet-induced inflammation in liver and adipose tissue, it is not effective in ameliorating obesity or obesity-linked metabolic disorders. Further studies are warranted to determine mechanisms by which COX-2 deletion impairs metabolic homeostasis.

## Data availability

Additional data will be shared upon request.

## Conflicts of interest

The authors declare that they have no conflicts of interest with the contents of this article.

## References

[1] Eskridge W., Cryer D.R., Schattenberg J.M., Gastaldelli A., Malhi H., Allen A.M. (2023). Metabolic dysfunction-associated steatotic liver disease and metabolic dysfunction-associated steatohepatitis: the patient and physician perspective. J. Clin. Med..

[2] Noureddin M. (2024). MASH clinical trials and drugs pipeline: an impending Tsunami. Hepatology.

[3] Harrison S.A., Bedossa P., Guy C.D., Schattenberg J.M., Loomba R., Taub R. (2024). A phase 3, randomized, controlled trial of Resmetirom in NASH with liver fibrosis. N. Engl. J. Med..

[4] Kuchay M.S., Choudhary N.S., Mishra S.K. (2020). Pathophysiological mechanisms underlying MAFLD. Diabetes Metab. Syndr..

[5] Duggan K.C., Walters M.J., Musee J., Harp J.M., Kiefer J.R., Oates J.A. (2010). Molecular basis for cyclooxygenase inhibition by the non-steroidal anti-inflammatory drug naproxen. J. Biol. Chem..

[6] Brune K., Patrignani P. (2015). New insights into the use of currently available non-steroidal anti-inflammatory drugs. J. Pain Res..

[7] Smith W.L., DeWitt D.L., Garavito R.M. (2000). Cyclooxygenases: structural, cellular, and molecular biology. Annu. Rev. Biochem..

[8] Basu S. (2007). Novel cyclooxygenase-catalyzed bioactive prostaglandin F2alpha from physiology to new principles in inflammation. Med. Res. Rev..

[9] Zhu G., Chen L., Liu S., She L., Ding Y., Yang C. (2022). Celecoxib-mediated attenuation of non-alcoholic steatohepatitis is potentially relevant to redistributing the expression of adiponectin receptors in rats. Heliyon.

[10] Chan P.C., Liao M.T., Hsieh P.S. (2019). The dualistic effect of COX-2-mediated signaling in obesity and insulin resistance. Int. J. Mol. Sci..

[11] Tans R., Bande R., van Rooij A., Molloy B.J., Stienstra R., Tack C.J. (2020). Evaluation of cyclooxygenase oxylipins as potential biomarker for obesity-associated adipose tissue inflammation and type 2 diabetes using targeted multiple reaction monitoring mass spectrometry. Prostaglandins Leukot. Essent. Fatty Acids.

[12] Murali G., Milne G.L., Webb C.D., Stewart A.B., McMillan R.P., Lyle B.C. (2012). Fish oil and indomethacin in combination potently reduce dyslipidemia and hepatic steatosis in LDLR(-/-) mice. J. Lipid Res..

[13] Zhang C., Lu Y., Song Y., Chen L., Hu J., Meng Y. (2022). Celecoxib attenuates hepatosteatosis by impairing de novo lipogenesis via Akt-dependent lipogenic pathway. J. Cell Mol. Med..

[14] Park S.Y., Cho W., Abd El-Aty A.M., Hacimuftuoglu A., Jeong J.H., Jung T.W. (2022). Valdecoxib attenuates lipid-induced hepatic steatosis through autophagy-mediated suppression of endoplasmic reticulum stress. Biochem. Pharmacol..

[15] Tsujimoto S., Kishina M., Koda M., Yamamoto Y., Tanaka K., Harada Y. (2016). Nimesulide, a cyclooxygenase-2 selective inhibitor, suppresses obesity-related non-alcoholic fatty liver disease and hepatic insulin resistance through the regulation of peroxisome proliferator-activated receptor gamma. Int. J. Mol. Med..

[16] Sinzinger H., Rogatti W. (1994). Prostaglandins and arterial wall lipid metabolism--in vitro, ex-vivo and in-vivo radioisotopic studies. J. Physiol. Pharmacol..

[17] Chan E.S., Zhang H., Fernandez P., Edelman S.D., Pillinger M.H., Ragolia L. (2007). Effect of cyclooxygenase inhibition on cholesterol efflux proteins and atheromatous foam cell transformation in THP-1 human macrophages: a possible mechanism for increased cardiovascular risk. Arthritis Res. Ther..

[18] Ekor M., Odewabi A.O., Kale O.E., Adesanoye O.A., Bamidele T.O. (2013). Celecoxib, a selective cyclooxygenase-2 inhibitor, lowers plasma cholesterol and attenuates hepatic lipid peroxidation during carbon-tetrachloride-associated hepatotoxicity in rats. Drug Chem. Toxicol..

[19] Ethridge R.T., Chung D.H., Slogoff M., Ehlers R.A., Hellmich M.R., Rajaraman S. (2002). Cyclooxygenase-2 gene disruption attenuates the severity of acute pancreatitis and pancreatitis-associated lung injury. Gastroenterology.

[20] Zhang M.Z., Yao B., Wang Y., Yang S., Wang S., Fan X. (2015). Inhibition of cyclooxygenase-2 in hematopoietic cells results in salt-sensitive hypertension. J. Clin. Invest..

[21] Keerthisingam C.B., Jenkins R.G., Harrison N.K., Hernandez-Rodriguez N.A., Booth H., Laurent G.J. (2001). Cyclooxygenase-2 deficiency results in a loss of the anti-proliferative response to transforming growth factor-beta in human fibrotic lung fibroblasts and promotes bleomycin-induced pulmonary fibrosis in mice. Am. J. Pathol..

[22] Adi N., Perriotte-Olson C., Desouza C.V., Ramalingam R., Saraswathi V. (2015). Hematopoietic cyclooxygenase-2 deficiency increases adipose tissue inflammation and adiposity in obesity. Obesity (Silver Spring).

[23] Huang J., Bathena S.P., Csanaky I.L., Alnouti Y. (2011). Simultaneous characterization of bile acids and their sulfate metabolites in mouse liver, plasma, bile, and urine using LC-MS/MS. J. Pharm. Biomed. Anal..

[24] Alnouti Y., Csanaky I.L., Klaassen C.D. (2008). Quantitative-profiling of bile acids and their conjugates in mouse liver, bile, plasma, and urine using LC-MS/MS. J. Chromatogr. B Analyt. Technol. Biomed. Life Sci..

[25] Bathena S.P., Mukherjee S., Olivera M., Alnouti Y. (2013). The profile of bile acids and their sulfate metabolites in human urine and serum. J. Chromatogr. B Analyt Technol. Biomed. Life Sci..

[26] Saraswathi V., Perriotte-Olson C., Ganesan M., Desouza C.V., Alnouti Y., Duryee M.J. (2017). A combination of dietary N-3 fatty acids and a cyclooxygenase-1 inhibitor attenuates nonalcoholic fatty liver disease in mice. J. Nutr. Biochem..

[27] Folch J., Lees M., Sloane Stanley G.H. (1957). A simple method for the isolation and purification of total lipides from animal tissues. J. Biol. Chem..

[28] Morrison W.R., Smith L.M. (1964). Preparation of fatty acid methyl esters and dimethylacetals from lipids with boron fluoride--methanol. J. Lipid Res..

[29] Vane J.R., Bakhle Y.S., Botting R.M. (1998). Cyclooxygenases 1 and 2. Annu. Rev. Pharmacol. Toxicol..

[30] Harris R.C. (2006). COX-2 and the kidney. J. Cardiovasc. Pharmacol..

[31] Wang C., Zhang X., Luo L., Luo Y., Wu D., Spilca D. (2022). COX-2 deficiency promotes white adipogenesis via PGE2-mediated paracrine mechanism and exacerbates diet-induced obesity. Cells.

[32] Narasimha A., Watanabe J., Lin J.A., Hama S., Langenbach R., Navab M. (2007). A novel anti-atherogenic role for COX-2--potential mechanism for the cardiovascular side effects of COX-2 inhibitors. Prostaglandins Other Lipid Mediat..

[33] Petit V., Arnould L., Martin P., Monnot M.C., Pineau T., Besnard P. (2007). Chronic high-fat diet affects intestinal fat absorption and postprandial triglyceride levels in the mouse. J. Lipid Res..

[34] Vegiopoulos A., Muller-Decker K., Strzoda D., Schmitt I., Chichelnitskiy E., Ostertag A. (2010). Cyclooxygenase-2 controls energy homeostasis in mice by de novo recruitment of brown adipocytes. Science.

[35] Zhang C.Y., Tan X.H., Yang H.H., Jin L., Hong J.R., Zhou Y. (2022). COX-2/sEH dual inhibitor alleviates hepatocyte senescence in NAFLD mice by restoring autophagy through Sirt1/PI3K/AKT/mTOR. Int. J. Mol. Sci..

[36] Liu C., Liu L., Zhu H.D., Sheng J.Q., Wu X.L., He X.X. (2018). Celecoxib alleviates nonalcoholic fatty liver disease by restoring autophagic flux. Sci. Rep..

[37] Voloshyna I., Kasselman L.J., Carsons S.E., Littlefield M.J., Gomolin I.H., De Leon J. (2017). COX-2-dependent and independent effects of COX-2 inhibitors and NSAIDs on proatherogenic changes in human monocytes/macrophages. J. Invest. Med..

[38] Reiss A.B., Anwar F., Chan E.S., Anwar K. (2009). Disruption of cholesterol efflux by coxib medications and inflammatory processes: link to increased cardiovascular risk. J. Invest. Med..

[39] Ouimet M., Franklin V., Mak E., Liao X., Tabas I., Marcel Y.L. (2011). Autophagy regulates cholesterol efflux from macrophage foam cells via lysosomal acid lipase. Cell Metab..

[40] Cawthon H., Chakraborty R., Roberts J.R., Backues S.K. (2018). Control of autophagosome size and number by Atg7. Biochem. Biophys. Res. Commun..

[41] Martinez-Lopez N., Singh R. (2015). Autophagy and lipid droplets in the liver. Annu. Rev. Nutr..

[42] Xu L., Liu W., Bai F., Xu Y., Liang X., Ma C. (2021). Hepatic macrophage as a key player in fatty liver disease. Front. Immunol..

[43] Kim B.M., Abdelfattah A.M., Vasan R., Fuchs B.C., Choi M.Y. (2018). Hepatic stellate cells secrete Ccl5 to induce hepatocyte steatosis. Sci. Rep..

[44] Borer J.S., Simon L.S. (2005). Cardiovascular and gastrointestinal effects of COX-2 inhibitors and NSAIDs: achieving a balance. Arthritis Res. Ther..

[45] Krotz F., Schiele T.M., Klauss V., Sohn H.Y. (2005). Selective COX-2 inhibitors and risk of myocardial infarction. J. Vasc. Res..

[46] Muscaritoli M., Anker S.D., Argiles J., Aversa Z., Bauer J.M., Biolo G. (2010). Consensus definition of sarcopenia, cachexia and pre-cachexia: joint document elaborated by Special Interest Groups (SIG) "cachexia-anorexia in chronic wasting diseases" and "nutrition in geriatrics". Clin. Nutr..

[47] Mariean C.R., Tiuca O.M., Mariean A., Cotoi O.S. (2023). Cancer cachexia: new insights and future directions. Cancers (Basel).

[48] Rogers E.S., MacLeod R.D., Stewart J., Bird S.P., Keogh J.W. (2011). A randomised feasibility study of EPA and Cox-2 inhibitor (Celebrex) versus EPA, Cox-2 inhibitor (Celebrex), resistance training followed by ingestion of essential amino acids high in leucine in NSCLC cachectic patients--ACCeRT study. BMC Cancer.

[49] Hu Y., Fu N., Chen L.X., Jiao J.H., Yang X.F. (2010). COX-2 regulates the proliferation and apoptosis of activated hepatic stellate cells through CDC27. J. Nanomater..

[50] Yu J., Hui A.Y., Chu E.S., Cheng A.S., Go M.Y., Chan H.L. (2007). Expression of a cyclo-oxygenase-2 transgene in murine liver causes hepatitis. Gut.

[51] Motino O., Agra N., Brea Contreras R., Dominguez-Moreno M., Garcia-Monzon C., Vargas-Castrillon J. (2016). Cyclooxygenase-2 expression in hepatocytes attenuates non-alcoholic steatohepatitis and liver fibrosis in mice. Biochim. Biophys. Acta.

